# Venous thromboembolism (VTE) prophylaxis in severely injured patients: an international comparative assessment

**DOI:** 10.1007/s00068-019-01208-z

**Published:** 2019-08-30

**Authors:** Amy C. Gunning, Ronald V. Maier, Doret de Rooij, Luke P. H. Leenen, Falco Hietbrink

**Affiliations:** 1 Department of Trauma Surgery, University Medical Center Utrecht, Suite: G04.228, Heidelberglaan 100, 3584 CX Utrecht, The Netherlands; 2 Department of Trauma Surgery, Harborview Medical Center, Seattle, USA

**Keywords:** Venous thromboembolism prophylaxis, Severely injured patients, International comparative assessment, Bleeding complications, Thromboembolic events

## Abstract

**Purpose:**

Venous thromboembolisms (VTE) are a major concern after acute survival from trauma. Variations in treatment protocols for trauma patients exist worldwide. This study analyzes the differences in the number of VTE events and the associated complications of thromboprophylaxis between two level I trauma populations utilizing varying treatment protocols.

**Methods:**

International multicenter trauma registry-based study was performed at the University Medical Center Utrecht (UMCU) in The Netherlands (early commencement chemical prophylaxis), and Harborview Medical Center (HMC) in the United States (restrictive early chemical prophylaxis). All severely injured patients (ISS ≥ 16), aged ≥ 18 years, and admitted in 2013 were included. Primary outcomes were VTE [deep venous thrombosis (DVT) (no screening), pulmonary embolism (PE)], and hemorrhagic complications.

**Results:**

In UMCU, 279 patients were included and in HMC, 974 patients. Overall, 75% of the admitted trauma patients in UMCU and 81% in HMC (*p* < 0.001) received thromboprophylaxis, of which 100% in and 75% at, respectively, UMCU and HMC consisted of chemical prophylaxis. From these patients, 72% at UMCU and 47% at HMC (*p* < 0.001) were treated within 48 h after arrival. At UMCU, 4 patients (1.4%) (PE = 3, DVT = 1) and HMC 37 patients (3.8%) (PE = 22, DVT = 16; *p* = 0.06) developed a VTE. At UMCU, a greater percent of patients with VTE had traumatic brain injuries (TBI). Most VTE occurred despite adequate prophylaxis being given (75% UMCU and 81% HMC). Hemorrhagic complications occurred in, respectively, 4 (1.4%) and 10 (1%) patients in UMCU and HMC (*p* = 0.570). After adjustment for age, ISS, HLOS, and injury type, no significant difference was demonstrated in UMCU compared to HMC for the development of VTE, OR 2.397, *p* = 0.102 and hemorrhagic complications, OR 0. 586, *p* = 0.383.

**Conclusions:**

A more early commencement protocol resulted in almost twice as much chemical prophylaxis being started within the first 48 h in comparison with a more delayed initiation of treatment. Interestingly, most episodes of VTE developed while receiving recommended prophylaxis. Early chemical thromboprophylaxis did not significantly increase the bleeding complications and it appears to be safe to start early.

## Introduction

Trauma is one of the leading causes of death and disability in every country of the world [1]. After the survival after the acute phase on the first day, the greatest concerns in these patients are life-threatening complications, such as venous thromboembolism (VTE) [2–5]. Pulmonary embolism is the third leading cause of death in trauma patients surviving the first day [3, 4], particularly in the severely injured patient [3, 5, 6]. The incidences of VTE in trauma patients are reported between 7 and 60% [3, 7], depending on the patient demographics, the methods of detection, and the type of prophylaxis [3, 4, 8]. Without prophylaxis, hospitalized patients following major trauma have a great risk of developing VTE [7, 9–12]. The increased risk of venous thrombosis in these patients is classically caused by endothelial injury, stasis of blood flow, and high intrinsic hypercoagulability, known as Virchow’s Triad [13, 14].

A recent Cochrane review showed that a combination of chemical prophylaxis, e.g., low molecular weight heparin (LMWH) [5, 7, 15], and mechanical prophylaxis, e.g., compression devices [13], are the most effective prevention for VTE (both DVT and PE) [6]. However, there is concern due to the associated potential increased hemorrhagic risk during LMWH treatment [15–18], in particular during surgery and shortly following traumatic brain injury (TBI) with intracranial bleeding. In fact, treatment is frequently considered contraindicated in patients with severe head trauma [19], because the bleeding risk is thought to outweigh the risk of VTE [11].

Worldwide, there is discrepancy between the thromboprophylaxis treatment protocols in trauma centers, especially in multi-trauma patients. However, direct comparisons have not been performed. This study evaluates the VTE events and the associated complications with thromboprophylaxis treatment in two severely injured trauma populations in two level I trauma centers in two different countries with different treatment protocols, a restrictive or delayed chemical thromboprophylaxis vs. an early commencement protocol.

## Methods

### Study design

This is an international multicenter trauma registry-based study performed at the University Medical Center Utrecht (UMCU) in the Netherlands, and Harborview Medical Center (HMC) in Seattle, Washington, the United States. Both trauma institutions are level I trauma centers functioning in a mature inclusive trauma system. In these trauma centers, adequate resources and personnel are available to provide care for every aspect of injury [20].

The Medical Ethics Committee of both institutions approved this study.

## University Medical Center Utrecht

**Fig. 1:**
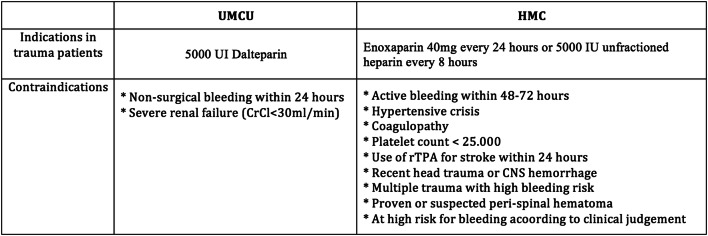
Indications and contraindications to pharmacologic prophylaxis

The UMCU is a level I trauma center located in Utrecht, the mid-region of The Netherlands. Four Levels II and III trauma centers are connected to this network. Annually, around 35,000 patients are admitted, of which ± 1300 are trauma patients and ± 375 are severely injured trauma patients [Injury Severity Score (ISS) ≥ 16]. According to the thromboprophylaxis protocol at UMCU, all admitted patients, aged ≥ 17, receive a subcutaneous injection of 2500 UI Dalteparin antiXa (Fragmin^®^, LMWH). Trauma patients are considered high-risk patients and receive daily a double dose or 5000 UI Dalteparin. Contraindications for chemical prophylaxis according to the hospital protocol are active bleeding on a non-surgical basis in the last 24 h, active endocarditis, severe renal failure (creatinine clearance rate < 30 ml/min), pregnancy and planned insertion or removal of an epidural catheter (Fig. 1) [21]. Post-discharge, thrombosis prophylaxis is indicated for 4 weeks in patients with a period of immobilization or with surgery of the hip or pelvis.

## Harborview Medical Center

Harborview Medical Center (HMC) is located in Seattle, Washington, in the United States (US), and admits annually around 19,000 patients. The level I trauma center receives approximately 6000 trauma admissions each year, of which over 2000 are severely injured patients (ISS ≥ 16). In this trauma center, according to the protocol, chemical prophylaxis, enoxaparin 40 mg (LMWH) every 24 h, or 5000 IU unfractionated heparin every 8 h, is indicated in trauma patients except with the following contraindications: active bleeding in the last 48–72 h, crisis, coagulopathy, platelet count < 25,000, used recombinant tissue plasminogen activator against stroke within 24 h, recent head trauma with CNS hemorrhage, multiple trauma with high bleeding risk, such as solid organ injury (suspected) peri-spinal hematoma, or at high risk for bleeding according to clinical judgment (Fig. 1). If chemical prophylaxis is contraindicated, sequential compression devices are used or an inferior vena cava filter (VCF), which is indicated in patients with a high risk for VTE, and when chemical prophylaxis is contraindicated [22].

## Patients

All severely injured patients, aged 18 years and older, and admitted at UMCU and HMC between January 1, 2013 and December 31, 2013 were included in the study. A severely injured patient was defined as a patient with an ISS ≥ 16. The ISS is a score based on the three highest, summed, and squared AIS scores of a patient. The AIS is a score, ranging from 1 (minor injury) to 6 (lethal injury) and represents the severity of the injury of a certain body region, i.e., head, face, thorax, abdomen, extremities, and external parts. We have excluded patients who were dead on arrival at the emergency department, patients transported to another hospital, and patients with missing data of their thromboprophylaxis treatment.

## Data

Data were collected from the institutional trauma registry and electronic medical records. The collected data for demographics were: age, gender, domestic use of anticoagulants or antiplatelet therapy, trauma mechanism, type of injury, TBI, hospital length of stay (HLOS), AIS score, and ISS.

The collected treatment data were: type of treatment, i.e., chemical prophylaxis or mechanical prophylaxis, and time between arrival at the hospital and first prophylaxis treatment, i.e., early prophylaxis ( ≤ 48 h after arrival) and late prophylaxis ( > 48 h after arrival).

Primary outcomes were venous thromboembolism (including all deep venous thrombosis in the body and pulmonary embolism) and hemorrhagic complications. Only patients who were clinically suspected for VTE were screened and eventually diagnosed. Complications were scored based on clinical findings and additional imaging. No routine imaging or screening for DVT detection was performed in either hospital. Secondary outcomes were hospital length of stay and mortality. Outcomes were specified for patients with TBI.

## Statistical analyses

Continuous variables were compared with independent sample Student’s *t *test and the Mann–Whitney *U* test. Categorical variables were compared with the chi-square test. Mean values are presented with their standard deviations (SD) and medians with their interquartile range (IQR). We have used multivariable logistic regression analyses to calculate the odds ratios (OR) for the development of VTEs and hemorrhagic complications in the two trauma populations. We have adjusted the OR for possible confounders, i.e., ISS, hospital LOS, and injury type. Other factors, such as body mass index and blood products, were considered but could not be included in the analysis as confounders due to the low number of events. Adjusting for more factors would lead to overadjusting and invalid outcomes.

All statistical analyses were performed with SPSS, version 20.0 (IBM Corp., Armonk, NY) for Windows. Significance of statistical differences was attributed to *p* < 0.05.

## Results

### Demographics

At UMCU, 330 severely injured patients were eligible for inclusion in the study. 51 patients were excluded due to transfers to another hospital during the post-traumatic course. In total, 279 patients were included for the analysis. In HMC, the data of thromboprophylaxis therapy began to be registered in the trauma registry from April 2013. Therefore, only patients admitted from April through December were included in this study; which included 974 severely injured patients eligible for analysis.

In both UMCU and HMC, the majority of patients were male (71.3% and 72.8%, respectively). Mean age was 52.9 at UMCU and 51.9 at HMC (*p* = 0.237). Significantly more penetrating trauma (10.0% vs. 1.4%, *p* < 0.001) and higher mean ISS (26.6 vs. 24.2, *p* < 0.001) were seen at HMC compared to UMCU. Overall, at UMCU, 75% of patients received some form of thromboprophylaxis during their admission compared to 81% at HMC (*p* ≤ 0.001). All patients at UMCU were treated with chemical thromboprophylaxis, none received a VCF. At HMC, 75% of the patients were treated with chemical prophylaxis, 12% were treated with a VCF of which 3% were treated with both chemical prophylaxis and a VCF. Seventy-two percent of the patients received chemical prophylaxis within the first 48 h after arrival at UMCU and 47% of the patients at HMC (*p* < 0.001). At UMCU, 4 patients (1.4%) developed a VTE [pulmonary embolism (PE) = 3, deep venous thrombosis (DVT) = 1], compared to 37 patients (3.8%) at HMC (PE = 22, DVT = 16; *p* = 0.06). Hemorrhagic complications occurred, respectively, in four (1.4%) and ten (1%) of patients at UMCU and HMC (*p* = 0.570). At HMC, nine patients with a VCF developed a VTE (PE = 5, DVT = 4) (8% of patients with VCF) after placement. All patients with a PE had a VCF at time of occurrence. At UMCU, three patients (75%) had a VTE while being treated with chemical prophylaxis. At HMC, 30 (81%) patients had a VTE while they were being treated with prophylaxis. The timing of the initiation in the patients with VTE ranged 2–14 at UMCU (mean 8.0 SD 5.9) and 0–18 at HMC (mean 2.2 SD 3.0) (*p* = 0.017).

In UMCU, one patient died after the development of PE and an intracranial hemorrhage 3 days later; this death could be marked as an attributable death. In HMC, two patients with a PE and DVT died; in these patients, these events were not considered as the cause of death. One patient in HMC died short after the admission due to a hemorrhage, however this patient did not receive any prophylaxis during the admission.

The patient characteristics are shown in Table 1.

**Table 1:** Patient characteristics

	UMCU	HMC
Patients, *n*	279	974
Age (mean)	53 (21)	52 (21)
Gender (male)	199 (71)	709 (73)
Injury type		
Blunt	272 (98)	831 (85)*
Penetrating	4 (1)	97 (10)*
Other	3 (1)	46 (5)
Injury Severity Score (mean)	24.2 (8.1)	26.6 (10.8)*
Hospital length of stay	10.0 (10–16)	9.0 (5.0–18.0)
ICU admission	101 (36)	935 (96)*
ICU length of stay	6.6 (7)	6.6 (8)
Anticoagulant (home use)	18 (7)	55 (6.0)*
Thromboprophylaxis (TP)	210 (75)	785 (81)*
Chemical TP (CTP)	210 (75)	734 (75)
Start CTP < 48 h	151 (72)	348 (38)*
Vena cava filter	0 (0)	117 (12)
Mortality	41 (15)	138 (14)
VTE	4 (1.4)	37 (3.8)
PE	3 (1.1)	22 (2.3)
DVT	1 (0.4)	16 (1.6)
Bleeding	4 (1.4)	10 (1.0)

*n*, number of patients (percentages)

^*^Significantly different

## Chemical thromboprophylaxis treatment and traumatic brain injury

The majority of the patients who did not receive chemical prophylaxis in the trauma populations had TBI, 93% and 84%, respectively, at UMCU and HMC. In total, 201 patients with TBI were admitted at UMCU and 622 at HMC. In both trauma centers, 70% of these patients received chemical prophylaxis. Respectively, 70 and 222 patients had isolated brain injury at UMCU and HMC, of which 57% in UMCU and 64% in HMC were overall treated with chemical prophylaxis. From all patients treated with chemical prophylaxis within 48 h of arrival, 19% in UMCU and 14% in HMC had an isolated injury of the brain. From all patients who did not receive chemical prophylaxis, 46% in UMCU and 37% in HMC were only injured to the brain. The majority of the patients in both hospitals received the chemical prophylaxis more than 48 h after their admission (Table 2).

**Table 2:** Distribution of chemical thromboprophylaxis (CTP) and complications in patients with traumatic brain injury (TBI)

	UMCU	HMC
TBI	201	622
TBI + treated with CTP*	141 (70)	435 (70)
Isolated TBI	70	222
Isolated TBI + treated with CTP^†^	40 (57)^†^	144 (64)^†^
Isolated TBI + treated with CTP < 48 h	13 (19)^‡^	30 (14)^‡^
Isolated TBI + VTE^a^	3 (4)	3 (1)
Isolated TBI + bleeding after CTP	2 (5)	3 (2)

*Percentages of total patients with TBI

^†^Percentages of total patients treated with CTP

^‡^Percentages of total patients with isolated TBI treated with CTP

^a^All patients except one patient in UMCU had CTP when the VTE event was diagnosed

## Adjusted outcomes

After adjustment for age, ISS, HLOS, and injury type, no significant difference was demonstrated in UMCU compared to HMC for the development of VTE, OR 2.397, *p* = 0.102 and hemorrhagic complications, OR 0.586, *p* = 0.383. Also, the VTE events further specified in DVT and PE did not show any significant difference between the two hospitals; PE, OR 1.818, *p* = 0.339 and DVT, OR 4.293, *p* = 0.160.

## Discussion

This study demonstrates the differences in number of VTE events and the associated complications with thromboprophylaxis between two level I trauma populations with different treatment protocols in two different countries. Although the number of patients who received thromboprophylaxis within 48 h after admission was significantly higher at UMCU, no significant difference was demonstrated in either the number of VTEs or hemorrhagic complications between the two populations. However, a noticeable trend with greater than twofold odds ratio was present with more delayed initiation of treatment.

There are still many uncertainties regarding the use of thromboprophylaxis in trauma patients, especially in severe trauma patients. On the one hand, therapy is indicated because it is demonstrated to decrease the risk of VTE, but on the other hand, it is contraindicated because of the potential hemorrhagic risks in these patients [6]. No clear indications exist concerning dosage, timing, frequency, and duration of prophylaxis. This allows variation in the thromboprophylaxis treatment protocols across similar leveled trauma centers, especially in patients who are frequently debated, those with TBI.

Several authors have argued caution in the use of thromboprophylaxis in trauma patients, because the bleeding risk might outweigh the risk of VTE, even more so in patients with TBI [11, 19]. Yet, other studies in addition to ours have demonstrated that the use of LMWH is safe in the majority of trauma patients, including patients with TBI after primary hemostasis has been accomplished or a delayed scan demonstrates a status quo antum [4]. Although in our study, significantly less patients with isolated TBI were treated with chemical prophylaxis, the bleeding rate was not significantly higher in patients with isolated TBI while under chemical thromboprophylaxis (Table 2). This suggests that the treatment is safe in the majority of these patients. Furthermore, in UMCU, the majority of the patients (75%) with a VTE also had isolated TBI. This differs from HMC, where only 8% of the patients with VTE had isolated TBI. We have no clear explanation for this; it could be related to the rare incidence of the events in both populations. However, these results suggest that patients with TBI might be more likely to develop VTEs, which is in line with the previous literature [3, 23–25] and might, therefore, benefit from early initiation of VTE prophylaxis.

Previous studies showed that the time of initiation of the chemical prophylaxis in patients is essential [26, 27]; a delay of > 4 days causes a threefold greater risk of VTE in major trauma patients [26]. In our study, while significantly more patients received prophylaxis within 48 h after admission at UMCU, the VTE rate did not significantly differ from the HMC population. This supports that the initiation time in the early stages is not a significant factor in the development of VTEs in these trauma populations or our study was underpowered to detect the difference. On the other hand, in accordance with other recent studies [28, 29], it appears safe to start chemical prophylaxis even at this early stage, even in patients with TBI [30, 31]. Furthermore, the timing of the initiation in the patients with VTE was significantly longer at UMCU. We should be careful to draw firm conclusions from this because the number of events, especially in UMCU, is very low and one outlier influences the mean drastically.

Interestingly, the vast majority of the patients who developed a VTE were adequately treated with chemical prophylaxis, 75% in UMCU and 81% in HMC. This suggests that patients developing a VTE are at such a high risk that even treatment with chemical prophylaxis is not sufficient or unable to be started early enough post-injury to demonstrate an effect.

Although this study demonstrates a low incidence of VTE in both level 1 centers with a well-established VTE prophylaxis protocol, these data question the validity of using VTE as a quality indicator, as proposed in the current literature [32–34], as most patients who developed a VTE were on adequate prophylaxis.

It is stated in the literature that trauma patients are at a high risk for the development of VTEs, in particular for DVTs. The incidence of DVT in the literature varies greatly between 11.8 and 65% [3, 35–37]. The incidence of PE is estimated between 1.5 and 20% [37–40]. A major cause of differences between these percentages and the percentages in our two populations in this study is thought to be due to differences in the manner of detection. In our study, no DVT screening was performed. The listed studies used pulmonary angiography, venography, and plethysmography in all patients [3, 35], while the outcome in our study was based on clinically detected and relevant VTEs. This has become a more commonly accepted approach. Our incidence rate corresponds with the German trauma population, which showed an incidence of 1.8% clinically relevant VTEs after severe trauma [12].

None of the patients in UMCU were treated with a VCF, compared to 117 (12%) patients in HMC. This is likely a consequence of the difference in treatment protocols. HMC maintains a protocol with more contraindications for chemical prophylaxis treatment and is, therefore, more likely to start with a different therapy in patients with a high risk of VTE. Still, 9 patients with a VCF developed a VTE compared to 108 patients with a VCF who did not develop a VTE. The high percentage of patients with a VCF and VTE development from the total number of patients with VTE (24%) could be due to the very high risk of VTE that already exists in these patients. Alternatively, it has been suggested that DVT and PE are two different entities in the trauma population as PE can develop without the presence of a DVT in the same patient [29, 41]. This is in contrast to the normal sequence seen in other patients where the PE is frequently associated with a standing DVT. The present study supports this as four patients developed a PE in the presence of a VCF. Therefore, the usefulness of a VCF becomes more debatable in trauma patients.

The results of this study might be limited for several reasons. One of the main reasons is the retrospective design of the study. The number of the events in our study was very low and could make it difficult to show a significant difference in the outcome in these populations. Furthermore, because of its retrospective design, the low incidence of VTE cannot directly be attributed to prophylaxis given. This study mainly focuses on chemical prophylaxis; still a fair number of patients were also treated with mechanical prophylaxis, such as compression devices. Unfortunately, these data were not sufficiently recorded and could not be included in this study. Furthermore, the potential impact of various different anticoagulants was not taken into account in this study.

According to the literature, multiple injuries increase the risk of VTE up to 60%. The results in the present study demonstrate that with a VTE prevention protocol in place, the incidence is reduced below 4%. No significant difference was demonstrated in the development of VTEs between two trauma populations treated in a similar level trauma center with different thromboprophylaxis treatment protocols with regard to the timing of initiation. Early initiation therapy appears safe, with respect to ongoing bleeding even in patients with TBI. Moreover, in concordance with recent reports, most VTEs developed under adequate prophylaxis, making VTEs not an adequate indicator for quality control measurements.
